# Revealing structure and shaping priorities in plant and fungal cell wall architecture via solid-state NMR

**DOI:** 10.1016/j.tcsw.2025.100159

**Published:** 2025-10-31

**Authors:** Peng Xiao, Priya Sahu, Sarah A. Pfaff, Ankur Ankur, Yohara K. Ranasinghe, Neil A.R. Gow, Jean-Paul Latgé, Daniel J. Cosgrove, Tuo Wang

**Affiliations:** a Department of Chemistry, Michigan State University, East Lansing, MI, USA; b Department of Biology, Pennsylvania State University, University Park, PA 16802, USA; c Medical Research Council Centre for Medical Mycology at the University of Exeter, University of Exeter, Geoffrey Pope Building, Stocker Road, Exeter EX44QD, UK; d Unité des Aspergillus, Institut Pasteur, Paris, France

**Keywords:** Cell wall, Fungi, Plant, Solid-state NMR, Polysaccharide, Antifungal, Xylan, Lignin, Chitin, Glucan

## Abstract

Plant and fungal cell walls are essential for growth, adaptation, and survival, with their intricate architectures dictating both resistance to stress and susceptibility to antifungal or biomass-degrading strategies. Understanding how these walls are built, remodeled, and function at the molecular level is therefore central to both clinical and biotechnological applications. Solid-state nuclear magnetic resonance (ssNMR) has emerged as a uniquely powerful tool for this purpose, as it reveals the structure, dynamics, and interactions of intact biopolymers without disrupting their native organization. Using this approach, recent studies have shown how structural polymorphism, polymer-polymer interactions, and species-specific remodeling govern mechanical integrity, drug resistance, and stress adaptation. Applications highlighted here include lignin-carbohydrate packing during plant stem maturation, fungal wall reorganization under treatment by wall-targeting antifungals such as echinocandin and nikkomycin, and the functional diversity of glucans, chitins, and mannans. Together, these insights uncover conserved principles of polymer assembly across kingdoms while informing new opportunities for antifungal development and biomass utilization. Ongoing advances in sensitivity and resolution are expected to broaden the reach of ssNMR and further accelerate its role in linking structural heterogeneity to biosynthetic complexity and biological function.

## Highlights

• Plant and fungal walls are dynamic, multi-component, and structurally complex
• SsNMR enables analysis of polymer dynamics and hydration in intact walls
• Fungal walls remodel under antifungals, maintaining rigidity and reducing water access
• Plant lignin selectively interacts with different polysaccharides, shaping wall mechanics
• Structural polymorphism links polymer function to function and biosynthesis

## 1 Introduction

Plant and fungal cell walls are essential extracellular matrices that provide structural support, mediate interactions with the environment, and serve as dynamic barriers against stress (Caffall and Mohnen, 2009; Chevalier et al., 2023; Cosgrove, 2024a; Dichtl et al., 2016; Hopke et al., 2018; J. P. Latgé, 2007). In plants, cell walls are traditionally described as composites of cellulose microfibrils embedded in hemicelluloses and pectic polymers in primary walls, or in hemicellulose and lignin in secondary lignified walls, all assembled through tightly regulated biosynthetic pathways (Cosgrove, 2024b; Anderson, 2015; Cosgrove, 2014; Zhong et al., 2018; Zhong and Ye, 2015). In fungi, chitin and glucans have been thought to form rigid scaffolds, decorated with mannans, glycoproteins, and other carbohydrate polymers, with biosynthesis controlled by enzyme families such as glucan synthases and chitin synthases (Free, 2013; Gow et al., 2017). While long recognized as crucial for growth and morphology, cell walls are also central to applied science: plant walls represent the largest terrestrial reservoir of renewable carbon for biofuel and biomaterial production (Rgauskas et al., 2014; Somerville et al., 2010), whereas fungal cell walls are unique in composition and thus serve as prime targets for antifungal therapy (Fisher et al., 2022; Gow, 2025; Odds et al., 2003). Together, these dual perspectives underscore the importance of understanding cell wall architecture and biosynthesis as both a fundamental biological question and a foundation for translational advances.

However, this task is particularly challenging because cell walls are both heterogeneous and complex, and further complicated by their dynamic nature, as they continuously remodel during growth and in response to stress (Cosgrove, 2024a; Gow, 2025; Gow and Lenardon, 2023; Wolf et al., 2012). Recently, our understanding of fungal and plant cell walls has been significantly advanced by the introduction of solid-state NMR (ssNMR) spectroscopy, which provides crucial structural information without disrupting the native state of the cell or its wall (Addison et al., 2025; Ghassemi et al., 2022; Munson et al., 2022; Reif et al., 2021; W. Zhao et al., 2022). This approach complements traditional chemical and imaging analyses, enabling a more comprehensive understanding of cell wall architecture and organization (J.P. Latgé and Wang, 2022).

It should be noted that ssNMR has been used in cell wall research for decades, primarily relying on one-dimensional ^13^C spectra to track resolved peaks of specific polymers (e.g., cellulose and chitin) and monitor changes in their content and structure within the cell wall (Atalla and Vanderhart, 1984; A. N. Fernandes et al., 2012; Foston, 2014; Heux et al., 2000; Sparrman et al., 2019; Thomas et al., 2013). Only recently has high-resolution ssNMR insight into cell wall organization become possible. This technical revolution rests on multiple technical advances, including the expansion of the pulse sequence library over the past decades and the adaptation of numerous high-resolution multidimensional correlation and spectral editing methods, primarily borrowed from protein NMR structural biology and polymer ssNMR, for carbohydrate characterization (Ladizhansky et al., 2025; Reif et al., 2021; Schmidt-Rohr and Mao, 2002; Shcherbakov et al., 2022; Williams et al., 2015). These advances, combined with the integration of sensitivity-enhancing techniques such as DNP (Maly et al., 2008), the widespread availability of high-field magnets for sufficient spectral resolution, and the utilization of less commonly used nuclei like ^1^H (Le Marchand et al., 2022), now provide a powerful and versatile toolbox capable of detailing the molecular structure of biomolecules and cell wall architecture (Chow et al., 2022; Ghassemi et al., 2022).

In this review, we envision the alignment of ssNMR studies with the priorities of fungal and plant cell wall research (Boerjan et al., 2024; Gow et al., 2023), highlighting the potential of this technique to elucidate the structural and chemical principles that govern polymer architecture, dynamics, and cell wall assembly. We summarize the working principles of ssNMR in resolving polymer dynamics and cell wall architecture, with the goal of promoting collaborative efforts to study these sophisticated and dynamic structures. We further selectively highlight two recent advances: first, the assessment of adaptive remodeling in the cell walls of pathogenic fungi, including *Aspergillu*s, *Candida*, and *Mucor* species, under the effect of wall-targeting antifungal agents, including both β-glucan and chitin inhibitors; and second, the temporal mapping of lignin-polysaccharide interactions in *Arabidopsis* inflorescence stems, resolving the structural roles of distinct xylan conformers and lignin units during stem maturation, as well as elucidating the contribution of methylated pectin in establishing lignin contact during early-stage lignification.

## 2 Biophysical insights provided by ssNMR into biopolymer structure, dynamics, and assembly

**Fig. 1:**
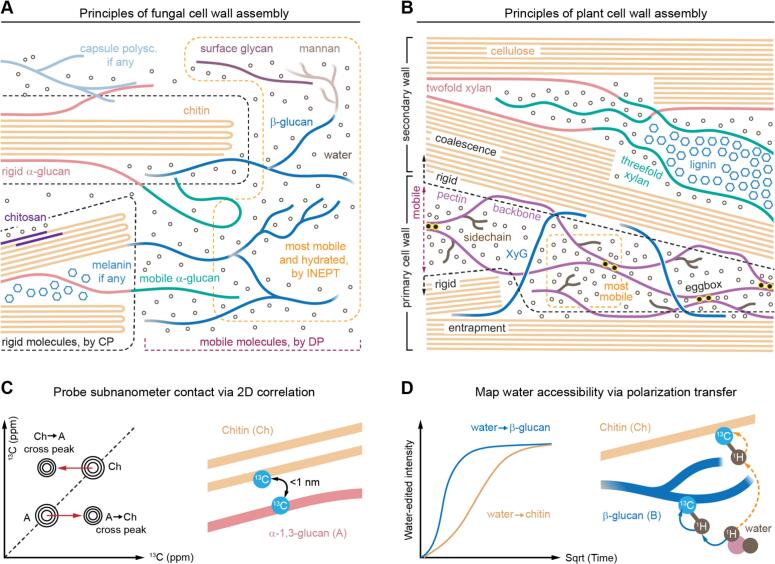
**Principles of fungal and plant cell wall assembly selectively visualized by ssNMR.** The structural principles governing polymer packing and supramolecular assembly, as revealed by ssNMR, are illustrated for (**A**) fungal cell walls and (**B**) plant primary and secondary cell walls. For chitin and cellulose, the number of strands depicted are only spacer holders and does not correspond to the actual number of chains within the microfibrils. In the fungal cell wall illustration, key ssNMR experiments highlight selective detection of rigid molecules (via CP), mobile molecules (via DP with short recycle delays), and highly mobile fractions (via INEPT), enclosed in dashed lines in black, purple, and yellow, respectively. In the plant cell wall illustration, dashed lines are used to separate three domains: highly mobile regions (yellow, mostly pectin sidechains and backbones), mobile regions (purple mostly matrix polysaccharides), and rigid regions (black; cellulose microfibrils and associated matrix polysaccharides in primary cell walls, and most molecules in secondary cell walls). The structural and physical principles underlying key experiments are illustrated for (**C**) the detection of intermolecular interactions and (**D**) the evaluation of water accessibility in cell wall polymers. (For interpretation of the references to colour in this figure legend, the reader is referred to the web version of this article.)

Across both naturally occurring cell walls in diverse organisms and artificially manufactured biomaterials, conserved structural principles govern the assembly of biopolymers. From the view of ssNMR, these systems are typically organized into several interrelated components: (i) a rigid scaffold that imparts mechanical strength and defines overall wall rigidity (black dashed region in Fig. 1A), (ii) a highly hydrated and soft matrix that permits the penetration and distribution of water and small molecules (yellow dashed region in Fig. 1A), and (iii) an intermediately mobile domain that bridges the rigid and soft regions, thereby facilitating structural integration and functional versatility (the transition region in Fig. 1A).

The rigid platform in naturally occurring cell walls includes crystalline chitin domains in fungi and cellulose microfibrils in plants. These rigid domains can exhibit varying degrees of association and flexibility. In plants, both hydrogen bonding and other noncovalent interactions contribute to the assembly of microfibrils (Jarvis, 2023; Wohlert et al., 2021). In fungi, chitin chains assemble into microfibrils and larger crystalline regions through diverse hydrogen-bonding patterns, most commonly antiparallel arrangements (Fig. 1A) (Fernando et al., 2021; Rinaudo, 2006). Matrix carbohydrates often decorate the surfaces of these rigid domains. While initially mobile, they may become rigidified through interactions with the crystalline core or become entrapped within it when multiple glucan chains hydrogen-bond to form microfibrils, when microfibrils aggregate, or when larger crystalline sites develop.

In plants, cellulose microfibrils can coalesce into larger bundles, in some cases termed macrofibrils, which are typically observed in secondary cell walls (Fig. 1B) (A. N. Fernandes et al., 2012; Lyczakowski et al., 2019; Terashima et al., 2009). Although coalescence can also occur in primary walls, it is less extensive. The mechanical properties of plant cell walls are strongly regulated by polymer-polymer interactions within the rigid core, such as fibril-fibril association and fibril sliding in primary walls (Zhang et al., 2021). Hemicelluloses, such as xyloglucan, may also become entrapped within or among cellulose microfibrils. This entrapment not only contributes to wall mechanics but also plays a key role in wall expansion, as the cellulose-xyloglucan may serve as a target for loosening proteins such as expansins (Cosgrove, 2024a; Park and Cosgrove, 2012).

The rigid core, as a structural platform, accommodates both covalent and non-covalent interactions with matrix carbohydrate polymers. Chemical analyses have shown that chitin can be covalently crosslinked to β-1,3-glucan (Gastebois et al., 2009; J. P. Latgé and Chamilos, 2019). In *Aspergillus*, this β-1,3-glucan is further connected to the larger matrix formed by branched β-1,3/1,6-glucan and galactomannan, whereas in *Candida* species, it is linked to β-1,6-glucan, which in turn connects the inner wall network to the outer domain composed of mannan fibrils and mannoproteins (Fontaine et al., 2000; Gow and Lenardon, 2023). SsNMR studies have additionally revealed that a substantial fraction of α-1,3-glucan is tightly packed against chitin surfaces, thereby becoming rigidified (Fig. 1A). This rigidified α-1,3-glucan, together with chitin and the covalently linked segments of β-glucans, forms the rigid core of the fungal cell wall, which can be selectively detected by dipolar-coupling-based cross polarization (CP) ssNMR experiments (Chakraborty et al., 2021; Kang et al., 2018; Schaefer and Stejskal, 1976).

The rigid α-1,3-glucan domains extend outward into the more mobile matrix, where they coexist with the bulk of β-glucans and other highly dynamic molecules. Regions distant from the chitin-based rigid core are well hydrated and remain highly mobile and can therefore be selectively detected by the J-coupling-based insensitive nuclei enhanced by polarization transfer (INEPT) experiment (Fig. 1A) (Elena et al., 2005). This experiment also reveals the presence of surface molecules and exopolysaccharides, such as galactosaminogalactan in *A. fumigatus*. Both the highly mobile matrix and the semi-mobile bridging domain are detectable by ^13^C direct polarization (DP) experiments with short recycle delays, which preferentially capture molecules with rapid ^13^C-T_1_ relaxation.

Chitosan contributes additional flexibility and water accessibility to the otherwise dehydrated and crystalline chitin domains. Melanin, when deposited, typically localizes in close proximity to the rigid core, where chitin and, as recently demonstrated by ssNMR in *C. neoformans*, α-glucans provide essential interactions for melanin packing (Ankur et al., 2025). The capsule also exhibits both rigid and mobile components: the rigid fraction arises from interactions with underlying cell wall polysaccharides, such as α-glucans in *C. neoformans* (Ankur et al., 2025; Lends et al., 2025).

In secondary plant cell walls, xylan adopts a twofold flat-ribbon conformation that coats the surface of cellulose microfibrils (Fig. 1B) (Simmons et al., 2016). Along with a portion of threefold xylan that is directly associated with cellulose, this forms part of the rigid domain. The remaining threefold xylan, which modulates water activity, retains its mobility (Kang et al., 2019). Threefold xylan also interacts with the hydrophobic nanodomains of lignin, making this conformer bi-functional (Kang et al., 2019). In primary plant cell walls, xyloglucan associates with cellulose microfibrils at discrete sites, either through entrapment or surface docking, and becomes rigidified in those regions (Park and Cosgrove, 2012). Portions of the pectin also establish extensive contacts with cellulose microfibrils and are thereby rigidified, whereas the rest of the pectin remain highly dynamic and function primarily to regulate water activity within the matrix (T. Wang et al., 2015).

As discussed above, ssNMR provides a versatile set of polarization methods that enable selective detection of rigid, mobile, and highly mobile fractions, as well as quantitative measurements (e.g., direct polarization with long recycle delays). This allows different components of the cell wall to be probed in situ, without the need for destructive isolation. Molecular dynamics can be quantified through measurements of NMR relaxation and dipolar couplings, which can be further converted into order parameters and correlation times characteristic of specific motions (Chevelkov et al., 2009; Tatman et al., 2025; Yarava et al., 2025). Intermolecular interactions are typically resolved using 2D and 3D correlation methods, where polarization transfer between distinct molecules can be detected (Delcourte et al., 2025; Hou et al., 2013; Shekar et al., 2022). For example, cross-peaks between chitin and α-glucan in correlation spectra reflect bidirectional polarization transfer, occurring both from chitin to α-glucan and from α-glucan to chitin, at the subnanometer scale (Fig. 1C). Water accessibility is assessed by ^1^H-polarization transfer from localized water to biomolecules, distinguishing well-hydrated species such as β-glucans from poorly hydrated ones such as chitin (Fig. 1D). The availability of this versatile ssNMR toolbox—together with ongoing methodological advances, such as ^1^H-detected experiments that complement conventional ^13^C-based approaches—is crucial for deepening our understanding of structurally and dynamically heterogeneous biocomposites (Xiao et al., 2025b; Bahri et al., 2023; Duan and Hong, 2024; Le Marchand et al., 2022; Lends et al., 2025; Yarava et al., 2025).

## 3 Positioning ssNMR studies within emerging priorities in plant and fungal cell wall research

Part of the motivation for writing this review is to align recent ssNMR studies with the top unresolved questions in fungal and plant cell wall structure and biology, as highlighted by prominent experts in the field in two recent *Cell Surface* Feature articles (Boerjan et al., 2024; Gow et al., 2023), and to explore future opportunities for bridging these important knowledge gaps.

SsNMR has long been employed in plant cell wall research, initially focusing on the structural characterization of cellulose (Atalla and Vanderhart, 1984; A. N. Fernandes et al., 2012; Foston, 2014; Sparrman et al., 2019). More recent high-resolution 2D and 3D ssNMR studies of intact plant cell walls have also contributed significantly to addressing several of the top five unanswered questions in plant cell surface research (Boerjan et al., 2024). Specifically, it provides structural insights into the structure-function relationships of cell wall polymers (Unanswered Question #1). A unique strength of ssNMR lies in its ability to analyze biopolymers within intact cell walls, where polymer structures are inherently polymorphic and differ from those in purified states due to a higher degree of conformational freedom and extensive intermolecular interactions present in the native cellular environment. A prominent example is the identification of at least seven distinct glucan environments in cellulose, corresponding to specific surface sites, including two deeply embedded sites and surface-adjacent interior sites, complicated further by hemicellulose binding and inter-fibrillar aggregation (Kang et al., 2019; Wang et al., 2016b). Another notable case is the differentiation of functional diversity between two-fold and three-fold xylan conformations, which preferentially associate with cellulose surfaces and lignin nanodomains, respectively; here, the terms ‘two-fold’ and ‘three-fold’ refer to distinct helical screw conformations of the xylan polymer (Grantham et al., 2017; Kirui et al., 2022; Simmons et al., 2016). Such structural features, along with their resulting functional selectivity, are often lost when polymers are purified for conventional chemical analyses but are fully preserved in native samples examined by ssNMR. Beyond these examples, ssNMR serves as a contributor to multiscale characterization of cell wall materials, often coupled with modeling methods, providing molecular-level structural information across length scales from angstroms to nanometers (Addison et al., 2024). To achieve a comprehensive model of the cell wall, it is often necessary to integrate ssNMR observations with a broad range of experimental data from other techniques, including, but not limited to, spectroscopic methods, microscopic analyses, and diffraction or scattering approaches (Bauer, 2012; Bhagia et al., 2022; Langan et al., 2014; Lyczakowski et al., 2019; Martinez-Sanz et al., 2015; Nicolas et al., 2022). Nonetheless, caution should be exercised when integrating these results with mesoscale and higher-level observations. It is important to note that while ssNMR detects extensive molecular interactions between polysaccharides, these interactions do not necessarily reflect the strength of binding or their mechanical implications. In particular, the observation of widespread cellulose-pectin contacts should not be interpreted as evidence of strong or load-bearing associations (Dick-Perez et al., 2011; Kirui et al., 2021; Phyo et al., 2017; T. Wang et al., 2015; T. Wang et al., 2012), but rather as an indication of spatial proximity within the cell wall matrix (Cosgrove, 2014).

SsNMR studies also shed light on cell wall architecture, clarifying how it is organized and assembled across species and cell types, and how these structures adapt during growth and under changing environments (Unanswered Question #2). Specifically, ssNMR gives critical information about the proximity of components of this wall and how this is modulated by imposed stretch. This approach benefits from the unique ability of ssNMR to pinpoint site-specific interactions between polymers, most often by mapping subnanometer-scale carbon‑carbon proximities. Beyond the recently resolved differences in binding partners of diverse xylan conformers, and further, their substitution patterns, in secondary cell walls, earlier ssNMR studies identified extensive cellulose-pectin colocalization and the functional binding sites of expansins in primary cell walls (Dick-Perez et al., 2011; T. Wang et al., 2015). In these cases, the non-lytic wall-loosening proteins facilitate wall expansion during plant growth by either disrupting cellulose-xyloglucan interactions in *Arabidopsis* or targeting the junctions between highly substituted and sparsely substituted glucuronoarabinoxylan (GAX) in grass species (Cosgrove, 2024a; T. Wang et al., 2013; Wang et al., 2016a). This technique has also been used to analyze the structural features of the egg-box formed by homogalacturonan calcium cross-linking in the cell wall, whose formation is regulated by the level of pectin methyl esterification, and to evaluate structural perturbations in the overall cell wall architecture (Temple et al., 2022). More recent studies have further resolved the dynamic evolution of the lignin-carbohydrate interface across developmental stages, a topic that will be highlighted later in this review (Xiao et al., 2025a). Environmental variables could also be incorporated into such analyses; however, their inclusion requires careful selection, as ssNMR measurements are inherently time- and cost-intensive and should be aligned with prioritized biological questions.

Beyond plant cell wall investigations, the past seven years have seen a marked increase in high-resolution ssNMR studies across diverse fungal species. These efforts have driven a paradigm shift in our understanding of fungal cell wall architecture and structural dynamics, particularly with respect to the molecular mechanisms underlying resistance to antifungal agents and adaptation to environmental stressors (Fernando et al., 2023a). Furthermore, this body of work has demonstrated the capacity of ssNMR to directly address all five of the major outstanding questions in fungal cell surface biology (Gow et al., 2023).

SsNMR has emerged as a critical tool for dissecting fungal cell wall architecture and dynamics in intact cells. It enables direct observation of biopolymers such as glucans, chitin, and mannoproteins, providing molecular-level insights into how walls remodel under environmental and pharmacological stresses, addressing the top unanswered question #1 of how fungal walls adapt to changing conditions. Notable applications include characterization of cell wall remodeling in *A. fumigatus*, *C. albicans*, and *C. auris* in response to β-1,3-glucan biosynthesis inhibitors such as echinocandins, as well as the assessment of nikkomycin's effects on chitin/chitosan-rich walls of *Rhizopus* and *Mucor* species (Cheng et al., 2024; Dickwella Widanage et al., 2024; Dickwella Widanage et al., 2025; I. Gautam et al., 2024). These studies highlight the potential of how ssNMR could possibly inform strategies for targeting polysaccharide synthesis with antifungals and vaccines (unanswered question #3). The same approach can be extended to evaluate responses to other cell-wall-targeting agents, including ibrexafungerp, fosmanogepix, and emerging compounds, thereby enabling structure-guided rational design of next-generation antifungals (Deng et al., 2025; C. M. Fernandes et al., 2022; Jallow and Govender, 2021; Maji et al., 2023; Shaw and Ibrahim, 2020).

SsNMR also provides unique insights into cell wall rigidity and permeability (unanswered question #2), key determinants of wall remodeling and adaptive survival. Across diverse fungal species, ssNMR consistently reveals reduced water accessibility and increased wall rigidity under stress, including antifungal exposure or extreme environmental conditions such as hypersalinity, temperature, and pH (Fernando et al., 2023b). These measurements are enabled by a versatile NMR toolbox, encompassing relaxation studies, dipolar couplings, chemical shift anisotropy, and exchange experiments, which collectively probe motions spanning nanoseconds to seconds. Water retention, reflecting cell wall permeability, can be quantified through polarization transfer from water molecules to tightly associated biopolymers, linking hydration dynamics directly to structural and functional changes in the wall (Fig. 1D).

Integrating the extensive ssNMR results across fungal species will ultimately enable the identification of universal structural principles that govern the assembly of carbohydrate molecules into larger composites, and ultimately, the cell wall. This directly addresses a component of unanswered question #4 by clarifying the roles specific polysaccharides play in assembly, where local rules determine the final architecture. Notable examples include the spacer function of α-glucans, which contribute both to the rigid core and the soft matrix, exhibiting a high degree of structural polymorphism that allows binding to various carbohydrate and non-carbohydrate polymers, and even forming rigid domains independently in the absence of chitin microfibrils (Ankur et al., 2025; Kang et al., 2018). Another key molecule is chitosan, which contributes to flexibility and water accessibility within crystalline domains originally formed solely by chitin—a feature important for restoring cell wall dynamics after initial rigidification in response to antifungal treatment (Dickwella Widanage et al., 2024; Mouyna et al., 2020). These structural functions have not been previously recognized, and delineating the fundamental roles of each carbohydrate is crucial for understanding how cell walls can be assembled under diverse conditions, stressed or unstressed, and across different morphotypes. (Fernando et al., 2022; Lamon et al., 2022; Singh et al., 2025).

SsNMR is a powerful tool for revealing how fungal cell walls interact with and respond to other microorganisms and environmental molecules, providing key insights into their role in shaping microbial relationships and community functions—the fifth unresolved questions in fungal cell surface research. A recent study of *Candida albicans* and *E. coli* cocultures revealed a decrease in β-1,3-glucan and β-1,6-glucan, an increase in mannan content, and reduced β-glucan branching in the fungus (Davis et al., 2025). These structural changes, detected by ssNMR, were correlated with increased surface exposure of both β-glucan and mannan following co-incubation with bacteria, as a response to bacterial metabolites. This, in turn, was associated with altered antifungal susceptibility and modulation of both innate and adaptive immune responses. A key feature of this study is the ability of ssNMR to resolve both fungal and bacterial carbohydrates in coculture, a capability that opens the door to investigating many other important questions related to microbial community interactions.

## 4 Dynamic remodeling of fungal cell walls for adaptive survival under antifungal stress

Fungal pathogens exhibit remarkable plasticity in their cell wall architecture, enabling them to adapt to environmental stresses, including exposure to antifungal agents. One of the most important contributions of ssNMR has been its ability to reveal, in unprecedented molecular detail, how different fungal species remodel their cell walls in response to antifungals, uncovering species-specific strategies that underline drug tolerance and resistance.

**Fig. 2:**
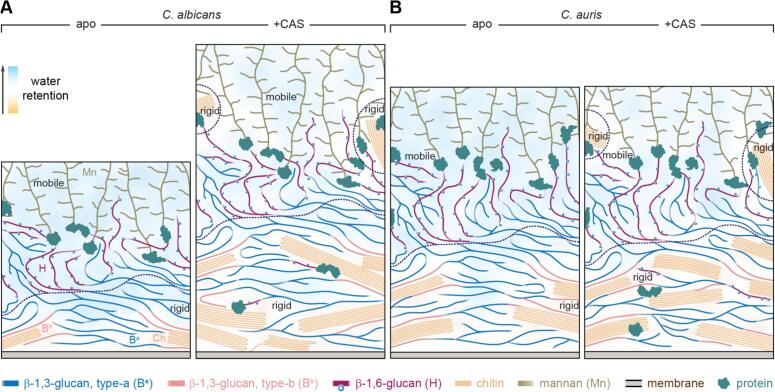
**Remodeling *Candida* cell walls by caspofungin.** Comparative views are provided for (**A**) *C. albicans* and (**B**) *C. auris* under control conditions (apo) and following caspofungin treatment (+CAS). Dashed lines distinguish rigid and mobile domains within the wall. Carbohydrates and other components are colour-coded. The background gradient from orange to blue indicates the hydration profile, with orange representing poorly hydrated regions and blue representing highly hydrated regions. Open blue circles are the β-1,3-glucoside sidechains of β-1,6-glucan. Adapted from (Dickwella Widanage et al., 2025), an open-access publication. (For interpretation of the references to colour in this figure legend, the reader is referred to the web version of this article.)

In a comparative study of *C. albicans* and the multidrug-resistant *C. auris*, ssNMR data demonstrated that, although both species share a conserved cell wall architecture, their responses to the β-1,3-glucan inhibitors named echinocandins (e.g., caspofungin, anidulafungin, micafungin and rezafungin) diverge significantly (Dickwella Widanage et al., 2025; Gow, 2025). Both species possess a simple two-component rigid core composed of chitin and part of β-1,3-glucan, embedded within a soft matrix of β-1,6-glucan, mannan, and the remaining portion of β-1,3-glucan (Fig. 2A, B). Upon treatment with sub-MIC (minimum inhibitory concentration) concentrations of the β-1,3-glucan inhibitor caspofungin, both species exhibited rigidification of certain β-1,6-glucan and *N*-mannan side chains due to newly formed interactions with chitin microfibrils, which were relocated from inner to outer domains. This reorganization, together with the removal of most β-1,3-glucan, the best-hydrated molecule in the fungal rigid core, resulted in reduced water accessibility.

However, differences emerged between the two species. Only *C. albicans* exhibited a 1.5-fold increase in cell wall thickness (Fig. 2A), while *C. auris* displayed more pronounced dehydration of its inner rigid core compared to *C. albicans* (Fig. 2B). In *C. albicans*, chitin became less flexible, but the remaining β-1,3-glucan retained higher dynamicity, revealing polymer separation and cell wall reorganization. In contrast, the rigid core of *C. auris* showed negligible changes in wall thickness, polymer dynamics, or intermolecular packing. Notably, the echinocandin-resistant strain I.3 maintained an identical cell wall composition after treatment. This structural inertness likely enables the strain to preserve essential cell wall functions required for resistance.

It was also observed that β-1,6-glucan is the molecule whose behavior differs between *C. albicans* and *C. auris*: caspofungin reduces its presence in the mobile fraction of the former but not the latter. The critical role of this carbohydrate in *C. auris* is further supported by the finding that deletion of the KRE6a gene, required for β-1,6-glucan synthesis, leads to decreased susceptibility to echinocandins (including both caspofungin and micafungin in this study) (Dickwella Widanage et al., 2025). Thus, ssNMR has identified β-1,6-glucan as the key carbohydrate that differentiates the cell walls of these two closely related species, despite their overall structural similarity. This result, consistent with recent biochemical studies, highlights the importance of understanding the structural role of β-1,6-glucan in *Candida* species—a long-overlooked molecule (Bekirian et al., 2024; Dickwella Widanage et al., 2025).

**Fig. 3:**
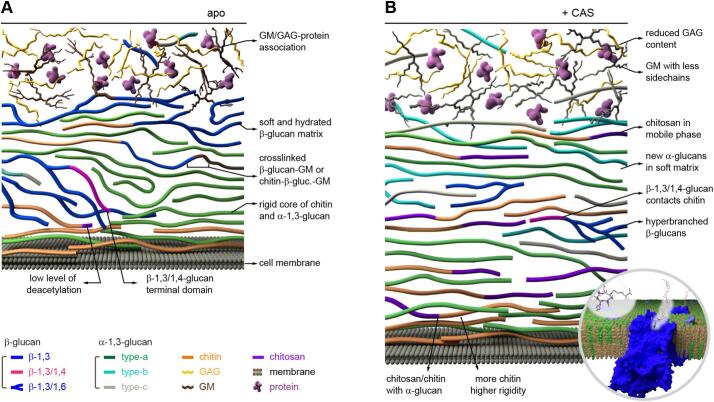
**Restructured *A. fumigatus* cell walls upon caspofungin exposure.** (**A**) Untreated *A. fumigatus* mycelial cell walls, composed of chitin, chitosan, α-1,3-glucans, β-1,3-glucans, and other polymers arranged outside the plasma membrane. Galactosaminogalactan: GAG; galactomannan: GM. (**B**) Caspofungin treatment fully restructures the cell wall, increasing thickness, altering carbohydrate composition, and enhancing intermolecular contacts. The inset highlights β-1,3-glucan synthase within the plasma membrane. Adapted from (Dickwella Widanage et al., 2024), an open-access publication.

In *A. fumigatus*, ssNMR revealed that the efficacy of echinocandins is constrained by compensatory cell wall remodeling (Dickwella Widanage et al., 2024). This process involves restructuring of molecules as well as dynamic rearrangements of mobile and rigid polymers, enabling the fungus to withstand antifungal stress (Fig. 3A, B). Beyond the expected inhibition of β-1,3-glucan synthesis, which was confirmed to occur within 12 h of drug exposure, the study revealed a complex and multistep remodeling of cell wall architecture that counteracts the drugs' effects. These changes to the mechanical core include an initial increase in chitin on day 1, which rigidifies the cell wall, followed by a rise in chitosan and highly polymorphic α-1,3-glucans on days 2–3, restoring flexibility. The rigid core of the cell wall exhibited decreased hydration, likely reducing permeability, limiting caspofungin diffusion, and enhancing fungal survival under antifungal stress. Further ssNMR analyses revealed that interactions between chitin and the buffering molecule α-1,3-glucans were strengthened in response to caspofungin treatment, a structural reinforcement likely critical for maintaining cell wall integrity despite the inhibition of β-1,3-glucan synthesis (Fig. 3B). β-glucans that survive echinocandin treatment are reorganized into a hyperbranched structure (Fig. 3B). These findings underscore the importance of considering the entire cell wall architecture when developing antifungal strategies, as the capacity of *A. fumigatus* to remodel its cell wall in response to echinocandin treatment highlights the need for therapies that target multiple components to effectively counter fungal infections.

Recently Baldus and colleagues used high-resolution ^1^H-detection ssNMR to understand how the host-defense peptide cathelicidin-2 (CATH-2) impacts the cell wall architecture of *A. fumigatus* (Bishoyi et al., 2025). They found that CATH-2 treatment disrupted the surface carbohydrate galactosaminogalactan and other components in the mobile matrix. Additionally, prolonged exposure to CATH-2 enhanced water accessibility in the otherwise hydrophobic rigid cell wall core, suggesting partial remodeling of the mechanical scaffold. These findings demonstrate the structural mechanism of how the peptide can penetrate the cell wall to reach the plasma membrane for function.

**Fig. 4:**
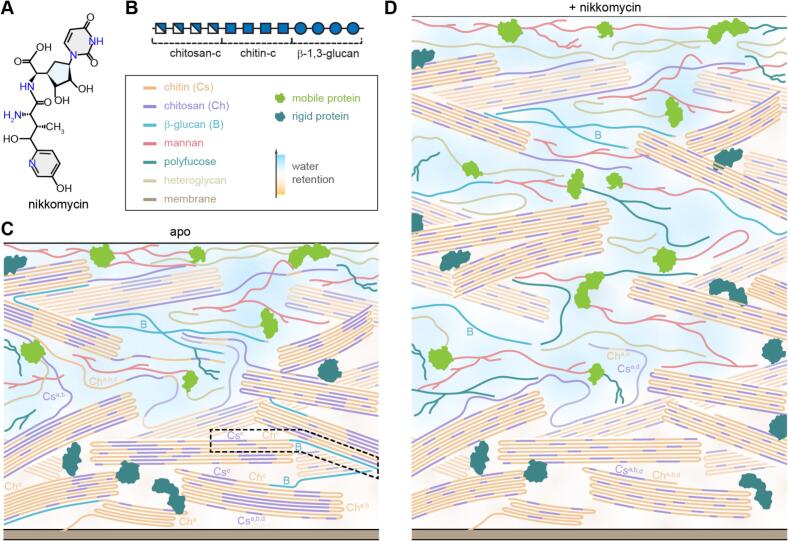
**Removal of a specific chitosan-chitin-glucan complex in *Rhizopus* by nikkomycin.** (**A**) Chemical structure of nikkomycin Z. (**B**) Schematic representation of the chitosan/chitin–β-glucan complex targeted and depleted by nikkomycin, illustrated using the Symbol Nomenclature for Glycans (SNFG). Models of *R. delemar* cell walls are shown for (**C**) untreated (apo) and (**D**) nikkomycin-treated conditions, highlighting changes in polymer organization and relative hydration. The dashed line region corresponds to the chitosan-chitin-β-glucan complex as illustrated in panel B, which will be depleted upon nikkomycin treatment. Carbohydrates, proteins, and hydration profiles are colour-coded as indicated in the accompanying legend box. Adapted from (Cheng et al., 2024), an open-access publication.

SsNMR analysis of *R. delemar* revealed that the rigid core of its chitin- and chitosan-dominated cell wall is primarily composed of highly polymorphic chitin and chitosan, with minimal β-glucans (Cheng et al., 2024). Chitosan is critical for maintaining hydration and dynamics, while some proteins are entrapped within the semi-crystalline chitin/chitosan layer, stabilized by hydrophobic residues and positioned away from β-glucans. Treatment with the chitin synthase inhibitor nikkomycin selectively removes a specific β-glucan-chitin-chitosan complex (Fig. 4A, B), leaving three other chitin and chitosan allomorphs intact, while simultaneously thickening and rigidifying the cell wall (Fig. 4C, D). This observation explains the limited efficacy of existing chitin inhibitors and highlights the importance of understanding the diverse families of chitin synthases and deacetylases to improve antifungal strategies targeting chitin- and chitosan-dominated species.

Understanding these species-specific cell wall remodeling strategies is essential for enhancing the efficacy of current antifungals and guiding the development of novel therapies. Building on these exploratory studies, future ssNMR investigations can be applied directly to resistant clinical isolates to provide precise structural insights into antifungal resistance mechanisms.

## 5 Evolution of lignin-carbohydrate interactions during plant stem maturation

**Fig. 5:**
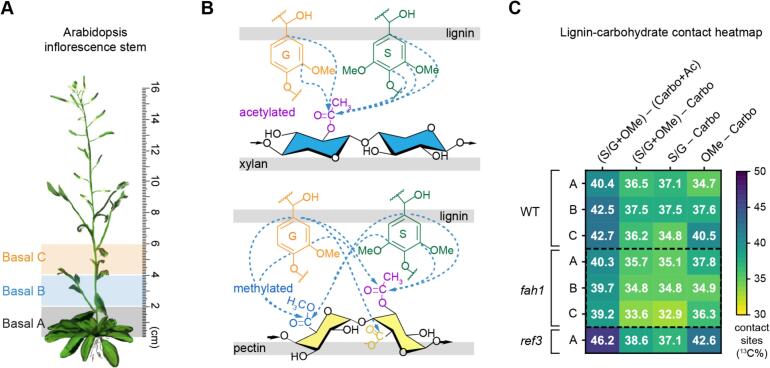
**Tracking lignin-carbohydrate interactions during *Arabidopsis* stem development.** (**A**) Illustration of an *Arabidopsis* inflorescence stem showing basal segments C, B, and A sampled for ssNMR analysis. (**B**) SsNMR-detected intermolecular interactions between lignin aromatic carbons and carbonyls in acetylated xylan (purple sites, top panel) or methylated GalA in homogalacturonan (blue sites, bottom panel). (**C**) Heatmap of carbon percentage at the lignin-carbohydrate interface across seven samples, including basal segments A-C for WT and *fah1* mutants, and basal segment A for the *ref3* mutant. Dashed boxes highlight weakened interactions observed in *fah1* sample, compared to WT and *ref3* samples across each single column. Figures are adapted from (Xiao et al., 2025a), an open-access publication. (For interpretation of the references to colour in this figure legend, the reader is referred to the web version of this article.)

Over the past decade, ssNMR has transformed our understanding of plant secondary cell walls by providing direct, molecular-level insights into their architecture and dynamics (Gao et al., 2020; Terrett et al., 2019). Building on this foundation, we recently applied high-resolution ^13^C-enriched ssNMR to *Arabidopsis thaliana* inflorescence stems, enabling detection of lignin-carbohydrate interactions across developmental stages (Fig. 5A) (Xiao et al., 2025a). The data revealed a progressive enrichment of syringyl (S) lignin in basal stem regions, and in older plants, where it becomes closely interwoven with carbohydrate scaffolds. Notably, acetylated xylan was identified as the primary carbohydrate partner of S-lignin, while guaiacyl (G) lignin exhibited preferential association with methylated pectin during early lignification (Fig. 5B). This monomer-specific binding highlights an unexpected degree of selectivity in lignin-polysaccharide interactions during plant maturation.

Mutant analyses further emphasized that the pattern of lignin-carbohydrate contact, rather than bulk lignin abundance, governs wall mechanics. The *fah1* mutant, deficient in S-lignin, displayed weakened lignin-carbohydrate interfaces (Fig. 5C) (Meyer et al., 1998; Xiao et al., 2025a). Conversely, the *ref3* mutant, despite having less total lignin, maintained robust wall integrity due to a favorable S/G ratio that preserved strong carbohydrate interactions (Fig. 5C) (Schilmiller et al., 2009; Xiao et al., 2025b). Together, these findings revealed that lignocellulosic performance arises not simply from lignin content but from its spatial and chemical integration with specific polysaccharides. Such mechanistic insights carry broad implications for biomass deconstruction strategies, crop engineering for resilience, and the rational design of lignocellulosic materials (Debnath et al., 2025).

## 6 Linking structural polymorphism to functional diversity and biosynthesis complexity

Structural polymorphism is a key feature of biomolecules in their native cellular state. Unlike isolated, purified, or crystallized molecules, biomolecules in intact cells can adopt a wide variety of conformations, exhibit heterogeneity in hydrogen bonding and other interactions, and display distinct dynamics. In ssNMR spectra collected from intact cells, this polymorphism is reflected by peak multiplicity, with each resolved signal representing a unique structural form of the same molecule. Early studies relied primarily on ^13^C–^13^C/^15^N 2D and 3D correlation experiments to resolve such features. For example, two- and three-fold xylans and seven distinct glucan chain forms in cellulose were resolved in plant cell walls, and a total of 45 chitin forms were identified across six fungal species (Fernando et al., 2021; Simmons et al., 2016).

Recent advances integrating ^1^H-detection with ^13^C spectra have further improved resolution of structural polymorphism, better resolving fine features of rigid molecules while producing solution-like spectra for mobile molecules, all within intact cells. This approach has resolved three forms of α-1,3-glucans in *A. fumigatus* and *A. nidulans*, three forms of β-1,3-glucan and eight forms of 1,2-linked mannan side chains in *C. albicans* and *C. auris*, and five forms of α-1,3-glucans in *C. neoformans* (Ankur et al., 2025; Dickwella Widanage et al., 2025; Isha Gautam et al., 2025). The advent of ultrahigh-field magnets (1.0–1.5 GHz NMR spectrometers) is expected to substantially increase the number of structural features that can be resolved in intact cells (S. Wang et al., 2025).

A key question remains as to why these structural forms matter and whether they can be correlated with structural function and biosynthetic complexity. The extreme polymorphism observed in chitin may reflect the large variety of chitin synthases (CHS), while the moderate polymorphism of α-1,3-glucan may, to some extent, correspond to the three major α-glucan synthase (AGS) proteins (Henry et al., 2012; Muszkieta et al., 2014). However, these connections require further ssNMR validation using deletion mutants.

Interactions within the cell wall also contribute to polymorphism. For example, in *Rhizopus* chitin/chitosan, only a single form out of four is crosslinked to β-1,3-glucan and is removable by nikkomycin treatment (Fig. 4C) (Cheng et al., 2024). Similarly, in chitin-low *C. neoformans*, five forms of α-1,3-glucan are critical for cell wall assembly, fulfilling distinct roles: aggregating to form the rigid platform, dispersing to form the dynamic matrix with β-glucans, packing with chitin microfibrils, interacting with capsular polysaccharide glucuronoxylomannan on the surface, and associating with melanin in the inner core (Ankur et al., 2025). For α-glucans, this functional diversity and dynamic heterogeneity is the primary driver of the observed structural polymorphism, alongside contributions from biosynthetic diversity.

**Fig. 6:**
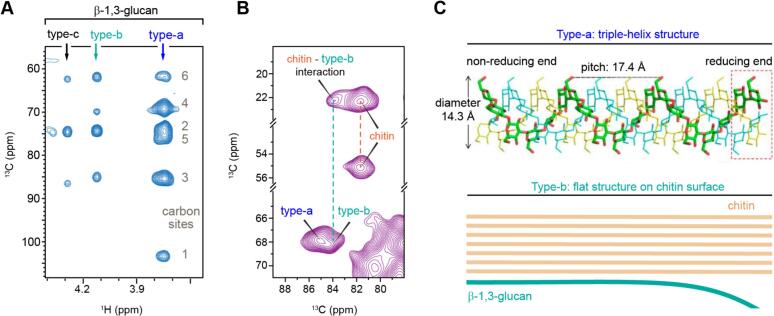
**Linking structural polymorphism to functional diversity.** (**A**) High-resolution ^1^H-detected spectrum of *C. auris* spectrum resolving three distinct β-1,3-glucan forms with unique chemical shifts. (**B**) DNP-enhanced 2D PAR spectra revealing intermolecular interactions between chitin and β-1,3-glucans; only type-b shows a cross peak with chitin. (**C**) Triple-helix model of β-1,3-glucan aligned with type-a chemical shifts; type-b arises from conformational changes induced by chitin microfibril interaction. Panels A and B are adapted from (Dickwella Widanage et al., 2025), an open-access publication. The top part of Panel C is adapted from (Manabe and Yamaguchi, 2021), an open-access publication.

The basis of β-1,3-glucan polymorphism has long been unclear, as only a single type of β-glucan synthase is present. Recent studies combining ^1^H-detection data, which resolve distinct forms (Fig. 6A), with Dynamic Nuclear Polarization (DNP) data, which reveal their interactions with other molecules (Fig. 6B), have provided new insights. In *C. auris*, three forms of β-1,3-glucan can be distinguished (Fig. 6A), in addition to the β-1,3,6-linked branching site (Dickwella Widanage et al., 2025). The bulk type-a signal perfectly correlates with the chemical shifts, and therefore the structure, of the triple-helix model of this polymer (Fig. 6C) (Manabe and Yamaguchi, 2021; Saito and Yokoi, 1989). In contrast, the minor type-b form interacts with chitin (Fig. 6B), forcing it to adapt to the surface of chitin crystalline domains and flattening its helical screw conformation (Fig. 6C) (Dickwella Widanage et al., 2025). This mechanism is analogous to the formation of two-fold xylan in plants, where it folds onto the flat surface of cellulose microfibrils from its three-fold helical structure in the matrix (Simmons et al., 2016). The only difference is that xylan requires evenly patterned acetylation or sidechains to stabilize its flat-ribbon structure (Grantham et al., 2017), whereas α-1,3-glucan does not. These observations suggest that the structural principles governing biopolymer assembly could be conserved across species and even across different organisms.

## 7 Conclusions and perspectives

SsNMR can characterize intact cell wall assemblies and architecture without extraction or chemical modification, preserving native interactions among biopolymers. This capability has revealed previously inaccessible features, including polysaccharide conformations, spatial proximities among wall polymers, and the progressive reorganization of wall networks. Consequently, ssNMR has become a central tool for understanding how structural heterogeneity contributes to mechanical function, stress adaptation, and biomass recalcitrance. Beyond its applications to fungal and plant cell walls highlighted in this selective review, this technique has also contributed conceptual advances to other extracellular matrices, including bacterial cell walls and biofilms, as well as algal carbohydrates and glycoproteins (Byeon et al., 2025; Jantschke et al., 2015; Poulhazan et al., 2024; Poulhazan et al., 2021; Reichhardt and Cegelski, 2014). Recent ssNMR investigations have even extended to understanding the digestion of plant cell walls by termites, the preservation and decomposition of plant cell walls into soil carbon, and the binding of insect cuticular proteins to chitin fibrils (Hu et al., 2025; Li et al., 2023; Xue et al., 2024, Xue et al., 2025; W. Zhao et al., 2025).

Despite these advances, a number limitations constrain the broader application of ssNMR. First, an important lesson we have learnt over the past few years is the value of working closely with experts in mycology and plant biology. As ssNMR data are biophysical and indirect, interpreting their biochemical significance requires contextualizing the results within decades of prior studies using other techniques. The findings do not always align. For example, we struggled to reconcile ssNMR-derived cell wall compositional changes with transcriptomic data in fungal studies, only to realize that transcriptomics reflects changes in gene expression, whereas ssNMR captures the final structural consequences, which are the result of the integration of many complex biological processes (Fernando et al., 2023b). Furthermore, different techniques may use different terminologies: a polymer described as being more dynamic may refer to entirely different motional characteristics depending on the method—for example, persistence length in polymer physics measured by neutron scattering compared to the more pronounced motion on a specific timescale measured by ssNMR relaxation. Addressing such discrepancies and appropriately integrating data from diverse transdisciplinary research disciplines through collaborative efforts of open-minded scientists remains a significant challenge.

Second, resolution can be limited in ssNMR. The structural similarity of monosaccharide units that make up cell wall polysaccharides makes achieving sufficient resolution challenging (W. Zhao et al., 2022). Overcoming this often requires the measurement of 2D or 3D correlation spectra involving ^13^C, ^15^N, ^1^H, and potentially ^17^O in the future, and may also benefit from the availability of additional ultrahigh-field magnets. Tailored polarization transfer pathways are typically needed to selectively detect cell wall molecules with specific structural motifs or unique dynamic characteristics. Even with these strategies, some carbohydrate components currently remain indistinguishable (W. Zhao et al., 2024). Therefore, resonance assignments—particularly when a species is being studied for the first time—often require validation using genetic mutants lacking specific carbohydrates and/or through biochemical analysis of cell wall composition.

Third, sensitivity can be low. This limitation arises from the inherently low sensitivity of NMR spectroscopy and the nature of the samples studied, where cellular extracts contain many different molecules, reducing the effective concentration of each type. Structural polymorphism further spreads the chemical shifts, diminishing signal intensity. Nevertheless, ssNMR is particularly powerful for investigating cell wall structure because polysaccharides consist of self-repeating units, which reduces the number of unique resonances and enhances the detectability of these repeated structures. However, minor linkages or low-abundance molecules (e.g., carbohydrate branching units and minor sidechains) sometimes remain undetectable, falling below the current sensitivity threshold. Efforts to improve ssNMR sensitivity through advances in DNP technology and more efficient bi-radicals, the development of solid-state cryoprobes (Hassan et al., 2020; Holmes et al., 2022; Vallet et al., 2024), as well as optimized ^1^H-detection tools for carbohydrate analysis (Bahri et al., 2023; Lends et al., 2025; Yarava et al., 2025), are expected to help overcome the sensitivity barrier.

Fourth, compared to other spectroscopic methods, ssNMR has relatively limited throughput. The combination of low sensitivity and complex data analysis makes it a time-intensive technique. Dedicated structural characterization can take multiple years and often requires a coordinated team of researchers. However, once the NMR fingerprint of a species is established, subsequent compositional analyses and rapid screening of polymer dynamics can be completed within a few weeks, making ssNMR a viable moderate throughput technique to complement other methods for cell wall analysis. In addition to ongoing improvements in sensitivity-enhancing technologies, the development of streamlined data-processing algorithms, aided by artificial intelligence (Cordova et al., 2022; Klukowski et al., 2023, Klukowski et al., 2025), is expected to alleviate this bottleneck, ultimately making the technique more accessible to the cell surface research community.

## CRediT authorship contribution statement

**Peng Xiao:** Conceptualization, Visualization, Writing – original draft. **Priya Sahu:** Conceptualization, Visualization, Writing – original draft. **Sarah A. Pfaff:** Writing – review & editing. **Ankur Ankur:** Writing – review & editing. **Yohara K. Ranasinghe:** Writing – review & editing. **Neil A.R. Gow:** Conceptualization, Writing – review & editing. **Jean-Paul Latgé:** Conceptualization, Writing – review & editing. **Daniel J. Cosgrove:** Conceptualization, Writing – review & editing. **Tuo Wang:** Conceptualization, Funding acquisition, Supervision, Writing – review & editing.

## Funding

The solid-state NMR analyses of plant secondary cell walls were supported by the 10.13039/100000015U.S. Department of Energy under grant no. DE-SC0023702 to T.W. The ssNMR work on fungal cell wall was primarily supported by the 10.13039/100000002National Institutes of Health (NIH) grant R01AI173270 to T.W. D.J.C was supported by the 10.13039/100000015U.S. Department of Energy under award no. DE-FG2-84ER13179 for work on expansins and wall mechanics. The work of S.P. on secondary cell wall formation was supported as part of the Center for Lignocellulose Structure and Formation, an Energy Frontier Research Center funded by the US Department of Energy, Office of Science, Basic Energy Sciences under award no. DE-SC0001090 to D.J.C. N.A.R.G. acknowledges support of Wellcome Trust Investigator, Collaborative, Equipment, Strategic and Biomedical Resource awards (101873, 200208, 215599, 224323). N.A.R.G. also thanks the MRC (MR/M026663/2) and the MRC Centre for Medical Mycology (MR/N006364/2) for support. This study/research is partly funded by the National Institute for Health and Care Research Exeter Biomedical Research Centre. The views expressed are those of the authors and not necessarily those of the NIHR or the Department of Health and Social Care.

## Declaration of competing interest

The authors declare the following financial interests/personal relationships which may be considered as potential competing interests: Tuo Wang reports financial support was provided by US Department of Energy. Tuo Wang reports financial support was provided by National Institutes of Health. Neil A.R. Gow reports financial support was provided by Wellcome Trust. Neil A.R. Gow reports financial support was provided by MRC Centre for Medical Mycology. Daniel J. Cosgrove reports financial support was provided by US Department of Energy. Neil A.R. Gow is an editor of The Cell Surface and has played no part in the handling of this submission. If there are other authors, they declare that they have no known competing financial interests or personal relationships that could have appeared to influence the work reported in this paper.

## Data Availability

No data was used for the research described in the article.

## References

[bb0005] Addison B., Bu L., Bharadwaj V., Crowley M.F., Harman-Ware A.E., Crowley M.F., Bomble Y.J., Ciesielski P.N. (2024). Atomistic, macromolecular model of the Populus secondary cell wall informed by solid-state NMR. Sci. Adv..

[bb0010] Addison B., Dickwella Widanage M.C., Pu Y., Ragauskas A.J., Harman-Ware A.E. (2025). Solid-state NMR at natural isotopic abundance for bioenergy applications. Biotechnol. Biofuels Bioprod..

[bb0015] Anderson C.T. (2015). We be jammin’: an update on pectin biosynthesis, trafficking and dynamics. J. Exp. Bot..

[bb0020] Ankur A., Yavara J.R., Gautam I., Scott F.J., Mentink-Vigier F., Chrissian C., Xie L., Roy D., Stark R.E., Doering T.L., Wang P., Wang T. (2025). Polymorphic α-glucans as structural scaffolds in Cryptococcus cell walls for chitin, capsule, and melanin: insights from 13C and 1H solid-state NMR. Angew. Chem. Int. Ed..

[bb0025] Atalla R.H., Vanderhart D.L. (1984). Native cellulose: a composite of two distinct crystalline forms. Science.

[bb0030] Bahri S., Safeer A., Adler A., Smedes H., van Ingen H., Baldus M. (2023). 1H-detected characterization of carbon–carbon networks in highly flexible protonated biomolecules using MAS NMR. J. Biomol. NMR.

[bb0035] Bauer S. (2012). Mass spectrometry for characterizing plant Cell Wall polysaccharides. Front. Plant Sci..

[bb0040] Bekirian C., Valsecchi I., Bachellier-Bassi S., Scandola C., Guijarro J.I., Chauvel M., Mourer T., Gow N.A.R., Aimanianda V., d’Enfert C., Fontaine T. (2024). Β-1,6-glucan plays a central role in the structure and remodeling of the bilaminate fungal cell wall. eLife.

[bb0045] Bhagia S., Durachko J., Lagana R., Kardosova M., Kacik F., Cernescu A., Schafer P., Yoo C.G., Ragauskas A.J. (2022). Nanoscale FTIR and mechanical mapping of plant cell walls for understanding biomass deconstruction. ACS Sustain. Chem. Eng..

[bb0050] Bishoyi A.K., van Neer J., Bahri S., Lorenz S., de Cock H., Baldus M. (2025). Solid-state NMR reveals reorganization of the aspergillus fumigatus Cell Wall due to a host-Defence peptide. Angew. Chem. Int. Ed..

[bb0055] Boerjan W., Burlat V., Cosgrove D.J., Dunand C., Dupree P., Haas K.T., Ingram G., Jamet E., Mohnen D., Moussu S., Peaucelle A., Persson S., Voiniciuc C., Hofte H. (2024). Top five unanswered questions in plant cell surface research. Cell Surf..

[bb0060] Byeon C.H., Kinney T., Saricayir H., Hansen K.H., Scott F.J., Sirinivasa S., Wells M.K., Mentink-Vigier F., Kim W., Akbey Ü. (2025). Ultrasensitive characterization of native bacterial biofilms via dynamic nuclear polarization-enhanced solid-state NMR. Angew. Chem. Int. Ed..

[bb0065] Caffall K.H., Mohnen D. (2009). The structure, function, and biosynthesis of plant cell wall pectic polysaccharides. Carbohydr. Res..

[bb0070] Chakraborty A., Fernando L.D., Fang W., Dickwella Widanage M.C., Wei P., Jin C., Fontaine T., Latgé J.P., Wang T. (2021). A molecular vision of fungal cell wall organization by functional genomics and solid-state NMR. Nat. Commun..

[bb0075] Cheng Q., Dickwella Widanage M.C., Yarava J.R., Ankur A., Latgé J.-P., Wang P., Wang T. (2024). Molecular architecture of chitin and chitosan-dominated cell walls in zygomycetous fungal pathogens by solid-state NMR. Nat. Commun..

[bb0080] Chevalier L., Pinar M., Borgne R.L., Durieu C., Penalva M.A., Boudaoud A., Minc N. (2023). Cell wall dynamics stabilize tip growth in a filamentous fungus. PLoS Biol..

[bb0085] Chevelkov V., Fink U., Reif B. (2009). Accurate determination of order parameters from ^1^H,^15^N dipolar couplings in MAS solid-state NMR experiments. J. Am. Chem. Soc..

[bb0090] Chow W.Y., De Papae G., Hediger S. (2022). Biomolecular and biological applications of solid-state NMR with dynamic nuclear polarization enhancement. Chem. Rev..

[bb0095] Cordova M., Engel E.A., Stefaniuk A., Paruzzo F., Hofstetter A., Ceriotti M., Emsley L. (2022). A machine learning model of chemical shifts for chemically and structurally diverse molecular solid. J. Phys. Chem. C.

[bb0100] Cosgrove D.J. (2014). Re-constructing our models of cellulose and primary cell wall assembly. Curr. Opin. Plant Biol..

[bb0105] Cosgrove D.J. (2024). Plant Cell Wall loosening by Expansins. Annu. Rev. Cell Dev. Biol..

[bb0110] Cosgrove D.J. (2024). Structure and growth of plant cell walls *Nat*. Rev. Mol. Cell Biol..

[bb0115] Davis F.A., Singh K., Krampen J.M., Bryant J.A., Ost K.S., Righi S.E., Balunas M.J., Wang T., R. O‘Meara (2025). Bacterial metabolites induce cell wall remodeling, antifungal resistance, and immune recognition of commensal fungi. BioRxiv.

[bb0120] Debnath D., Sahu P., Nejad M., Pu Y., Tessonnier J.P., Ragauskas A., Qi L., Wang T. (2025). Structure-guided utilization of lignocellulose for catalysis, energy, and biomaterials. Cell Rep. Phys. Sci..

[bb0125] Delcourte L., Berbon M., Rodriguez M., Delhaes L., Habenstein B., Loquet A. (2025). Solid-state NMR observation of chitin in whole cells by indirect 15N detection with NC, NCC, CNC and CNCC polarization transfers. Solid State Nucl. Magn. Reson..

[bb0130] Deng Q., Li Y., He W., Chen T., Liu N., Ma L., Qiu Z., Shang Z., Wang Z. (2025). A polyene macrolide targeting phospholipids in the fungal cell membrane. Nature.

[bb0135] Dichtl K., Samantaray S., Wagener J. (2016). Cell wall integrity signalling in human pathogenic fungi. Cell. Microbiol..

[bb0140] Dick-Perez M., Zhang Y., Hayes J., Salazar A., Zabotina O.A., Hong M. (2011). Structure and interactions of plant Cell-Wall polysaccharides by two- and three-dimensional magic-angle-spinning solid-state NMR. Biochemistry.

[bb0145] Dickwella Widanage M.C., Gautam I., Sarkar D., Mentink-Vigier F., Vermass J.V., Ding S.Y., Lipton A.S., Fontaine T., Latgé J.P., Wang P., Wang T. (2024). Adaptative survival of aspergillus fumigatus to echinocandins arises from cell wall remodeling beyond β−1,3-glucan synthesis inhibition. Nat. Commun..

[bb0150] Dickwella Widanage M.C., Singh K., Li J., Yarava J.R., Scott F.J., Xu Y., Gow N.A.R., Mentink-Vigier F., Wang P., Lamoth F., Wang T. (2025). Distinct echinocandin responses of Candida albicans and Candida auris cell walls revealed by solid-state NMR. Nat. Commun..

[bb0155] Duan P., Hong M. (2024). Selective detection of intermediate-amplitude motion by solid-state NMR. J. Phys. Chem. B.

[bb0160] Elena B., Lesage A., Steuernagel S., Bockmann A., Emsley L. (2005). Proton to Carbon-13 INEPT in solid-state NMR spectroscopy. J. Am. Chem. Soc..

[bb0165] Fernandes A.N., Thomas L.H., Altaner C.M., Callow P., Forsynth V.T., Apperley D.C., Kennedy C.J., Jarvis M.C. (2012). Nanostructure of cellulose microfibrils in spruce wood. Proc. Natl. Acad. Sci. USA.

[bb0170] Fernandes C.M., Normile T.G., Fabri J.H.T.M., Brauer V.S., Araujo G.R., Frases S., Nimrichter L., Malavazi I., Del Poeta M. (2022). Vaccination with live or heat-killed aspergillus fumigatus ΔsglA conidia fully protects immunocompromised mice from invasive aspergillosis. mBio.

[bb0175] Fernando L.D., Dickwella Widanage M.C., Penfield J., Lipton A.S., Washton N., Latgé J.P., Wang P., Zhang L., Wang T. (2021). Structural polymorphism of chitin and chitosan in fungal cell walls from solid-state NMR and principal component analysis. Front. Mol. Biosci..

[bb0180] Fernando L.D., Dickwella Widanage M.C., Shekar S.C., Mentink-Vigier F., Wang P., Wi S., Wang T. (2022). Solid-state NMR analysis of unlabeled fungal cell walls from aspergillus and Candida species. J. Struct. Biol. X.

[bb0185] Fernando L.D., Pérez-Llano Y., Widanage M.C.D., Jacob A., Martínez-Avila L., Lipton A.S., Gunde-Cimerman N., Latgé J.P., Batista-García R.A., Wang T. (2023). Structural adaptation of fungal cell wall in hypersaline environment. Nat. Commun..

[bb0190] Fernando L.D., Zhao W., Gautam I., Ankur A., Wang T. (2023). Polysacchride assemblies in fungal and plant cell walls explored by solid-state NMR. Structure.

[bb0195] Fisher M.C., Alastruey-Izquierdo A., Berman J., Bicanic T., Bignell E.M., Bowyer P., Bromley M.J., Bruggemann R., Garber G., Cornely O.A., Gurr S.J., Harrison T.S., Kuijper E., Rhodes J., Sheppard D.C., Warris A., White P.L., Xu J., Zwaan B., Verweij P.E. (2022). Tackling the emerging threat of antifungal resistance to human health. Nat. Rev. Microbiol..

[bb0200] Fontaine T., Simenel C., Dubreucq G., Adam O., Delepierre M., Lemoine J., Vorgias C.E., Diaquin M., Latgé J.P. (2000). Molecular organization of the alkali-insoluble fraction of aspergillus fumigatus cell wall. J. Biol. Chem..

[bb0205] Foston M. (2014). Advances in solid-state NMR of cellulose. Curr. Opin. Biotechnol..

[bb0210] Free S.J. (2013). Fungal cell wall organization and biosynthesis *Adv*. Genet.

[bb0215] Gao Y., Lipton A.S., Wittmer Y., Murray D.T., Mortimer J.C. (2020). A grass-specific cellulose-xylan interaction dominates in sorghum secondary cell walls. Nat. Commun..

[bb0220] Gastebois A., Clavaud C., Aimanianda V., Latgé J.-P. (2009). Aspergillus fumigatus: cell wall polysaccharides, their biosynthesis and organization. Future Microbiol..

[bb0225] Gautam I., Singh K., Dickwella Widanage M.C., Yarava J.R., Wang T. (2024). New vision of cell walls in aspergillus fumigatus from solid-state NMR spectroscopy. J. Fungi.

[bb0230] Gautam I., Yarava J.R., Xu Y., Li R., Scott F.J., Mentink-Vigier F., Momany M., Latgé J.-P., Wang T. (2025). Comparative analysis of polysaccharide and cell wall structure in aspergillus nidulans and aspergillus fumigatus by solid-state NMR. Carbohydr. Polym..

[bb0235] Ghassemi N., Poulhazan A., Deligey F., Mentink-Vigier F., Marcotte I., Wang T. (2022). Solid-state NMR investigations of extracellular matrixes and cell walls of algae, Bacteria, Fungi, and plants. Chem. Rev..

[bb0240] Gow N.A.R. (2025). Fungal cell wall biogenesis: structural complexity, regulation and inhibition. Fungal Genet. Biol..

[bb0245] Gow N.A.R., Lenardon M.D. (2023). Architecture of the dynamic fungal cell wall. Nat. Rev. Microbiol..

[bb0250] Gow N.A.R., Latgé J.P., Munro C.A. (2017). The fungal Cell Wall: structure, biosynthesis, and function. Microbiol. Spectr..

[bb0255] Gow N.A.R., Casadevall A., Fang W. (2023). Top five unanswered questions in fungal cell surface research. Cell Surf..

[bb0260] Grantham N.J., Wurman-Rodrich J., Terrett O.M., Lyczakowski J.J., Stott K., Iuga D., Simmons T.J., Durand-Tardif M., Brown S.P., Dupree R., Busse-Wicher M., Dupree P. (2017). An even pattern of xylan substitution is critical for interaction with cellulose in plant cell walls. Nat Plants.

[bb0265] Hassan A., Quinn C.M., Struppe J., Sergeyev I.V., Zhang C., Guo C., Runge B., Theint T., Dao H.H., Jaroniec C.P., Berbon M., Lends A., Habenstein B., Loquet A., Kuemmerle R., Perrone B., Gronenborn A.M., Polenova T. (2020). Sensitivity boosts by the CPMAS CryoProbe for challenging biological assemblies. J. Magn. Reson..

[bb0270] Henry C., Latgé J.P., Beauvais A. (2012). Alpha-1,3 glucans are dispensable in aspergillus fumigatus. Eukaryot. Cell.

[bb0275] Heux L., Brugnerotto J., Desbrieres J., Versali M.F., Rinaudo M. (2000). Solid state NMR for determination of degree of acetylation of chitin and chitosan. Biomacromolecules.

[bb0280] Holmes J.B., Liu V., Gaulkins B.G., Hilario E., Ghosh R.K., Drago V.N., Young R.P., Romero J.A., Gill A.D., Bogie P.M., Paulino J., Wang X.G.R., Bosken Y.K., Struppe J., Hassan A., Guidoulianov J., Perrone B., Mentink-Vigier F., Chang C.A., Long J., Hooley R., Mueser T.C., Dunn M., Mueller L.J. (2022). Imaging active site chemistry and protonation states: NMR crystallography of the tryptophan synthase α-aminoacrylate intermediate. Proc. Natl. Acad. Sci. USA.

[bb0285] Hopke A., Brown A.J.P., Hall R.A., Wheeler R.T. (2018). Dynamic fungal cell wall architecture in stress adaptation and immune evasion. Trends Microbiol..

[bb0290] Hou G., Yan S., Trébosc J., Amoureux J.-P., Polenova T. (2013). Broadband homonuclear correlation spectroscopy driven by combined R2_n_^v^ sequences under fast magic angle spinning for NMR structural analysis of organic and biological solids. J. Magn. Reson..

[bb0295] Hu S., Li J., Yuan F., Zhang J., Cheng X., Xiang S., Tian. (2025). Structural mechanism of insect Cuticular protein binding to chitin revealed by solid-state NMR. J. Am. Chem. Soc..

[bb0300] Jallow S., Govender N.P. (2021). Ibrexafungerp: A first-in-class Oral triterpenoid glucan synthase inhibitor. J. Fungi.

[bb0305] Jantschke A., Koers E., Mance D., Weingarth M., Brunner E., Baldus M. (2015). Insight into the supramolecular architecture of intact diatom biosilica from DNP-supported solid-state NMR spectroscopy. Angew. Chem. Int. Ed..

[bb0310] Jarvis M.C. (2023). Hydrogen bonding and other non-covalent interactions at the surfaces of cellulose microfibrils. Cellulose.

[bb0315] Kang X., Kirui A., Muszynski A., Widanage M.C.D., Chen A., Azadi P., Wang P., Mentink-Vigier F., Wang T. (2018). Molecular architecture of fungal cell walls revealed by solid-state NMR. Nat. Commun..

[bb0320] Kang X., Kirui A., Dickwella Widanage M.C., Mentink-Vigier F., Cosgrove D.J., Wang T. (2019). Lignin-polysaccharide interactions in plant secondary cell walls revealed by solid-state NMR. Nat. Commun..

[bb0325] Kirui A., Du J., Zhao W., Barnes W., Kang X., Anderson C.T., Xiao C., Wang T. (2021). A pectin methyltransferase modulates polysaccharide dynamics and interactions in Arabidopsis primary cell walls: evidence from solid-state NMR. Carbohydr. Polym..

[bb0330] Kirui A., Zhao W., Deligey F., Yang H., Kang X., Mentink-Vigier F., Wang T. (2022). Carbohydrate-aromatic interface and molecular architecture of lignocellulose. Nat. Commun..

[bb0335] Klukowski P., Riek R., Guntert P. (2023). Time-optimized protein NMR assignment with an integrative deep learning approach using AlphaFold and chemical shift prediction. Sci. Adv..

[bb0340] Klukowski P., Riek R., Guntert P. (2025). Machine learning in NMR spectroscopy. Prog. Nucl. Magn. Reson. Spectrosc..

[bb0345] Ladizhansky V., Palani R.S., Mardini M., Griffin R.G. (2025). Dipolar recoupling in rotating solids. Chem. Rev..

[bb0350] Lamon G., Lends A., Valsecchi I., Wong S.S.W., Dupres V., Lafont F., Tolchard J., Schmitt C., Mallet A., Grelard A., Morvan E., Dufourc E.J., Habenstein B., Guijarro J.I., Aimanianda V., Loquet A. (2022). Solid-state NMR molecular snapshots of aspergillus fumigatus cell wall architecture during a conidial morphotype transition. Proc. Natl. Acad. Sci. USA.

[bb0355] Langan P., Petridis L., O’Neill H.M., Pingali S.V., Foston M., Nishiyama Y., Schulz R., Lindner B., Hanson B.L., Harton S., Heller W.T., Urban V., Evans B.R., Gnanakaran S., Ragauskas A.J., Smith J.C., Davison B.H. (2014). Common processes drive the thermochemical pretreatment of lignocellulosic biomass. Green Chem..

[bb0360] Latgé J.P. (2007). The cell wall: a carbohydrate Armour for the fungal cell *Mol*. Microbiol.

[bb0365] Latgé J.P., Chamilos G. (2019). Aspergillus fumigatus and aspergillosis in 2019 *Clin*. Microbiol. Rev..

[bb0370] Latgé J.P., Wang T. (2022). Modern biophysics redefines our understanding of fungal Cell Wall structure complexity, and dynamics. mBio.

[bb0375] Le Marchand T., Schubeis T., Bonaccorsi M., Paluch P., Lalli D., Pell A.J., Andreas L.B., Jaudzems K., Stanek J., Pintacuda G. (2022). ^1^H-detected biomolecular NMR under fast magic-angle spinning. Chem. Rev..

[bb0380] Lends A., Lamon G., Delcourte L., Surny-Leclere A., Grelard A., Morvan E., Abdul-Shukkoor M.B., Berbon M., Vallet A., Habenstein B., Dufourc E.J., Schanda P., Aimanianda V., Loquet A. (2025). Molecular distinction of Cell Wall and capsular polysaccharides in encapsulated pathogens by in situ magic-angle spinning NMR techniques. J. Am. Chem. Soc..

[bb0385] Li H., Kang X., Yang M., Kasseney B.D., Zhou X., Liang S., Zhang X., Wen J.L., Yu B., Liu N., Zhao Y., Mo J., Currie C.R., Ralph J., Yelle D.J. (2023). Molecular insights into the evolution of woody plant decay in the gut of termites. Sci. Adv..

[bb0390] Lyczakowski J.J., Bourdon M., Terrett O.M., Helariutta Y., Wightman R., Dupree P. (2019). Structural imaging of native Cryo-preserved secondary cell walls reveals the presence of macrofibrils and their formation requires Normal cellulose, lignin and Xylan biosynthesis *front*. Plant Sci..

[bb0395] Maji A., Soutar C.P., Zhang J., Lewandowska A., Uno B.E., Yan S., Shelke Y., Murhade G., Nimerovsky E., Borcik C.G., Arango A.S., Lange J.D., Marin-Toledo J.P., Lyu Y., Bailey K.L., Andes D.R., Pogorelov T.V., Schwieters C.D., Fan T.M., Rienstra C.M., Burke M.D. (2023). Tuning sterol extraction kinetics yields a renal-sparing polyene antifungal. Nature.

[bb0400] Maly T., Debelouchina G.T., Bajaj V.S., Hu K.N., Joo C.G., Mak-Jurkauskas M.L., Sirigiri J.R., van der Wel P.C.A., Herzfeld J., Temkin R.J., Griffin R.G. (2008). Dynamic nuclear polarization at high magnetic fields. J. Chem. Phys..

[bb0405] Manabe N., Yamaguchi Y. (2021). 3D structural insights into β-glucans and their binding proteins. Int. J. Mol. Sci..

[bb0410] Martinez-Sanz M., Gidley M.J., Gilbert E.P. (2015). Application of X-ray and neutron small angle scattering techniques to study the hierarchical structure of plant cell walls: A review. Carbohydr. Polym..

[bb0415] Meyer K., Shirley A.M., Cusumano J.C., Bell-Lelong D.A., Chapple C. (1998). Lignin monomer composition is determined by the expression of a cytochrome P450-dependent monooxygenase in Arabidopsis. Proc. Natl. Acad. Sci. USA.

[bb0420] Mouyna I., Delliere S., Beauvais A., Gravelat F., Snarr B., Lehoux M., Zacharias C., Sun Y., Carrion S.D.J., Pearlman E., Sheppard D.C., Latgé J.P. (2020). What are the functions of chitin deacetylases in aspergillus fumigatus?. Front. Cell. Infect. Microbiol..

[bb0425] Munson C.R., Gao Y., Mortimer J.C., T., M. D. (2022). Solid-state nuclear magnetic resonance as a tool to probe the impact of mechanical preprocessing on the structure and arrangement of plant Cell Wall polymers. Front. Plant Sci..

[bb0430] Muszkieta L., Aimanianda V., Mellado E., Gribaldo S., Alcazar-Fuoli L., Szewczyk E., Prevost M.C., Latgé J.P. (2014). Deciphering the role of the chitin synthase families 1 and 2 in the in vivo and in vitro growth of aspergillus fumigatus by multiple gene targeting deletion. Cell. Microbiol..

[bb0435] Nicolas W.J., Faßler F., Dutka P., Schur Florian K.M., Jensen G., Meyerowitz E. (2022). Cryo-electron tomography of the onion cell wall shows bimodally oriented cellulose fibers and reticulated homogalacturonan networks. Curr. Biol..

[bb0440] Odds F.C., Brown A.J.P., Gow N.A.R. (2003). Antifungal agents: mechanisms of action. Trends Microbiol..

[bb0445] Park Y.B., Cosgrove D.J. (2012). A revised architecture of primary cell walls based on biomechanical changes induced by substrate-specific endoglucanases. Plant Physiol..

[bb0450] Phyo P., Wang T., Xiao C., Anderson C.T., Hong M. (2017). Effects of pectin molecular weight changes on the structure, dynamics, and polysaccharide interactions of primary cell walls of Arabidopsis thaliana: insights from solid-state NMR. Biomacromolecules.

[bb0455] Poulhazan A., Dickwella Widanage M.C., Muszynski A., Arnold A., Warschawsi D., Parastoo A., Isabelle M., Wang T. (2021). Identification and quantification of glycans in whole cells: architecture of microalgal polysaccharides described by solid-state NMR. J. Am. Chem. Soc..

[bb0460] Poulhazan A., Arnold A., Mentink-Vigier F., Muszynski A., Azadi P., Halim A., Vakhrushev S.Y., Joshi H.J., Wang T., Warschawsi D., Marcotte I. (2024). Molecular-level architecture of Chlamydomonas reinhardtii’s glycoprotein-rich cell wall. Nat. Commun..

[bb0465] Reichhardt C., Cegelski L. (2014). Solid-state NMR for bacterial biofilms. Mol. Phys..

[bb0470] Reif B., Ashbrook S.E., Emsley L., Hong M. (2021). Solid-state NMR spectroscopy. Nat. Rev. Methos Primers.

[bb0475] Rgauskas A.J., Beckham G.T., Biddy M.J., Chandra R., Chen F., Davis M.F., Davison B.H., Dixon R.A., Gilna P., Keller M., Langan P., Naskar A.K., Saddlere J.N., TSchaplinski T.J., Tuskan G.D., Wyman C.E. (2014). Lignin valorization: improving lignin processing in the biorefinery. Science.

[bb0480] Rinaudo M. (2006). Chitin and chitosan: properties and applications. Prog. Polym. Sci..

[bb0485] Saito H., Yokoi M. (1989). High-resolution ^13^C NMR study of (1->3)-beta-D-glucans in the solid state: DMSO-induced conformational change and conformational characterization by spin relaxation measurements. Bull. Chem. Soc. Jpn..

[bb0490] Schaefer J., Stejskal E.O. (1976). Carbon-13 nuclear magnetic resonance of polymers spinning at the magic angle. J. Am. Chem. Soc..

[bb0495] Schilmiller A.L., Stout J., Weng J.K., Humphreys J., Ruegger M.O., Chapple C. (2009). Mutations in the cinnamate 4-hydroxylase gene impact metabolism, growth and development in Arabidopsis. Plant J..

[bb0500] Schmidt-Rohr K., Mao J.D. (2002). Efficient CH-group selection and identification in ^13^C solid-state NMR by dipolar DEPT and ^1^H chemical-shift filtering. J. Am. Chem. Soc..

[bb0505] Shaw K.J., Ibrahim A.S. (2020). Fosmanogepix: A review of the first-in-class broad Spectrum agent for the treatment of invasive fungal infections. J. Fungi.

[bb0510] Shcherbakov A.A., Medeiros-Silva J., Tran N., Gelenter M.D., Hong M. (2022). From angstroms to nanometers: measuring interatomic distances by solid-state NMR. Chem. Rev..

[bb0515] Shekar S.C., Zhao W., Fernando L.D., Hung I., Wang T. (2022). A three-dimensional 13C-13C-13C DQ-SQ-SQ correlation experiment for high-resolution analysis of complex carbohydrates using solid-state NMR. J. Magn. Reson..

[bb0520] Simmons T.J., Mortimer J.C., Bernardinelli O.D., Poppler A.C., Brown S.P., deAzevedo E.R., Dupree R., Dupree P. (2016). Folding of xylan onto cellulose fibrils in plant cell walls revealed by solid-state NMR. Nat. Commun..

[bb0525] Singh K., Henry C., Mouyna I., Beauvais A., Xu Y., Karai A., van Rhijn V., Latgé J.P., Wang T. (2025). Kre6-dependent β-1,6-glucan biosynthesis only occurs in the conidium of aspergillus fumigatus. mSphere.

[bb0530] Somerville C., Youngs H., Taylor C., Davis S.C., Long S.P. (2010). Feedstocks for lignocellulosic biofuels. Science.

[bb0535] Sparrman T., Svenningsson L., Sahlin-Sjovold K., Nordstierna L., Westman G., Bernin D. (2019). A revised solid-state NMR method to assess the crystallinity of cellulose. Cellulose.

[bb0540] Tatman B.P., Sridharan V., Uttarkabat M., Jaroniec C.P., Ernst M., Rovo P., Schanda P. (2025). Bumps on the road: the way to clean relaxation dispersion magic-angle spinning NMR. J. Am. Chem. Soc..

[bb0545] Temple H., Phyo P., Yang W., Lyczakowski J.J., Echevarria-Poza A., Yakunin I., Parra-Rojas J.P., Terrett O.M., Saez-Aguayo S., Dupree R., Orellana A., Hong M., Dupree P. (2022). Golgi-localized putative S-adenosyl methionine transporters required for plant cell wall polysaccharide methylation. Nat Plants.

[bb0550] Terashima N., Kitano K., Kojima M., Yoshida M., Yamamoto H., Westermark U. (2009). Nanostructural assembly of cellulose, hemicellulose, and lignin in the middle layer of secondary wall of ginkgo tracheid. J. Wood Sci..

[bb0555] Terrett O.M., Lyczakowski J.J., Yu L., Iuga D., Franks W.T., Brown S.P., Dupree R., Dupree P. (2019). Molecular architecture of softwood revealed by solid-state NMR. Nat. Commun..

[bb0560] Thomas L.H., Forsyth V.T., Sturcova A., Kennedy C.J., May R.P., Altaner C.M., Apperley D.C., Wess T.J., Jarvis M.C. (2013). Structure of cellulose microfibrils in primary cell walls from collenchyma. Plant Physiol..

[bb0565] Vallet A., Ayala I., Perrone B., Hassan A., Simorre J.P., Bougault C., Schanda P. (2024). MAS NMR experiments of corynebacterial cell walls: complementary ^1^H- and CPMAS CryoProbe-enhanced ^13^C-detected experiments. J. Magn. Reson..

[bb0570] Wang T., Zabotina O.A., Hong M. (2012). Pectin–cellulose interactions in the arabidopsis primary cell wall from two-dimensional magic-angle-spinning solid-state nuclear magnetic resonance. Biochemistry.

[bb0575] Wang T., Park Y.B., Caporini M.A., Rosay M., Zhong L., Cosgrove D.J., Hong M. (2013). Sensitivity-enhanced solid-state NMR detection of expansin’s target in plant cell walls. Proc. Natl. Acad. Sci. USA.

[bb0580] Wang T., Park Y.B., Cosgrove D.J., Hong M. (2015). Cellulose-pectin spatial contacts are inherent to never-dried Arabidopsis thaliana primary cell walls: evidence from solid-state NMR. Plant Physiol..

[bb0585] Wang T., Chen Y., Tabuchi A., Cosgrove D.J., Hong M. (2016). The target of expansin EXPB1 in maize cell walls from binding and solid-state NMR studies. Plant Physiol..

[bb0590] Wang T., Yang H., Kubicki J.D., Hong M. (2016). Cellulose structural polymorphism in plant primary cell walls investigated by high-field 2D solid-state NMR spectroscopy and density functional theory calculations. Biomacromolecules.

[bb0595] Wang S., Ravula T., Stringer J.A.P.L.G.K., Warmuth O.A., Williams C.G., Thome A.F., Mueller L.J., Rienstra C.M. (2025). Ultrahigh-resolution solid-state NMR for high–molecular weight proteins on GHz-class spectrometers. Sci. Adv..

[bb0600] Williams J.K., Schmidt-Rohr K., Hong M. (2015). Aromatic spectral editing techniques for magic-angle-spinning solid-state NMR spectroscopy of uniformly 13C-labeled proteins *solid state Nucl*. Magn. Reson..

[bb0605] Wohlert M., Benselfelt T., Wagberg L., Furo I., Berglund L.A., Wohlert J. (2021). Cellulose and the role of hydrogen bonds: not in charge of everything. Cellulose.

[bb0610] Wolf S., Hematy K., Hofte H. (2012). Growth control and cell wall signaling in plants. Annu. Rev. Plant Biol..

[bb0615] Xiao P., Pfaff S., Zhao W., Debnath D., Vojvodin C.S., Liu C.J., Cosgrove D., Wang T. (2025). Emergence of lignin-carbohydrate interactions during plant stem maturation visualized by solid-state NMR. Nat. Commun..

[bb0620] Xiao P., Yarava J.R., Debnath D., Sahu P., Xu Y., Xie L., Holmes D., Wang T. (2025). Rapid high-resolution analysis of polysaccharide-lignin interactions in secondary plant cell walls using proton-detected solid-state NMR. Anal. Chem..

[bb0625] Xue Y., Li H., Kang X. (2024). Molecular unraveling of polysaccharide digestion in wood-feeding termites: A solid-state NMR perspective. Carbohydr. Polym..

[bb0630] Xue Y., Li H., Kang X. (2025). Arabinan-rich architectures in pectin Rhamnogalacturonan I domain unveiled by termite digestion: in situ structural insights from solid-state NMR. Carbohydr. Polym..

[bb0635] Yarava J.R., Gautam I., Jacob A., Fu R., Wang T. (2025). Proton-detected solid-state NMR for deciphering structural polymorphism and dynamic heterogeneity of cellular carbohydrates in pathogenic Fungi. J. Am. Chem. Soc..

[bb0640] Zhang Y., Yu J., Wang X., Durachko D.M., Zhang S., Cosgrove D.J. (2021). Molecular insights into the complex mechanics of plant epidermal cell walls. Science.

[bb0645] Zhao W., Deligey F., Shekar S.C., Mentink-Vigier F., Wang T. (2022). Current limitations of solid-state NMR in carbohydrate and cell wall research. J. Magn. Reson..

[bb0650] Zhao W., Debnath D., Gautam I., Fernando L.D., Wang T. (2024). Charting the solid-state NMR signals of polysaccharides: A database-driven roadmap. Magn. Reson. Chem..

[bb0655] Zhao W., Thomas E.C., Debnath D., Scott F.J., Mentink-Vigier F., White J.R., Cook R.L., Wang T. (2025). Enriched molecular-level view of saline wetland soil carbon by sensitivity-enhanced solid-state NMR. J. Am. Chem. Soc..

[bb0660] Zhong R., Ye Z.H. (2015). Secondary cell walls: biosynthesis, patterned deposition and transcriptional regulation. Plant Cell Physiol..

[bb0665] Zhong R., Cui D., Ye Z. (2018). Secondary cell wall biosynthesis. New Phytol..

